# Inosine-5′-monophosphate interacts with the TAS1R3 subunit to enhance sweet taste detection

**DOI:** 10.1016/j.fochms.2025.100246

**Published:** 2025-02-11

**Authors:** Christine Belloir, Lucie Moitrier, Adeline Karolkowski, Nicolas Poirier, Fabrice Neiers, Loïc Briand

**Affiliations:** Centre des Sciences du Goût et de l'Alimentation, CNRS, INRAE, Institut Agro, Université Bourgogne Europe, F-21000 Dijon, France

**Keywords:** G-protein-coupled receptor, Taste receptor, Sweetener, Umami, Ribonucleotide

## Abstract

Umami and sweet taste detection is mediated by the activation of the TAS1R1/TAS1R3 and TAS1R2/TAS1R3 receptors, respectively. TAS1R2-Venus flytrap domain (VFT) constitutes the primary ligand-binding site for most of the sweeteners whereas TAS1R1-VFT contains the orthosteric binding site for umami compounds. Inosine-5′-monophosphate (IMP), previously known to potentiate umami taste, binds to a site of TAS1R1-VFT adjacent to the L-glutamate site leading to umami synergy. However, the involvement of the TAS1R3 subunit in umami receptor-ligand interactions or in synergy with IMP has never been demonstrated. To elucidate the VFT contribution to umami and sweet detection, we expressed human TAS1R1- and TAS1R3-VFTs in bacteria. Ligand binding studies quantified by intrinsic tryptophan fluorescence revealed that both TAS1R1- and TAS1R3-VFTs are able to interact with umami compounds. Cellular assays revealed that IMP is able, like cyclamate, to modulate the response of TAS1R2/TAS1R3 and TAS1R3 alone stimulated by calcium ions. IMP also acted as an enhancer of TAS1R2/TAS1R3 when stimulated with sucralose, neotame and cyclamate. Taking together, our data demonstrated that IMP modulates sweet compound detection at the receptor level acting via the TAS1R3 subunit. This research suggests more complex receptor interactions between umami and sweet taste qualities and paves the way for development of new sweetness enhancers.

## Highlights

• TAS1R1 and TAS1R3 Venus flytrap domains interacted with L-glutamate and inosine-5′-monophosphate (IMP).
• IMP had a synergistic effect on L-glutamate and L-aspartate binding.
• IMP modulated responses of TAS1R2/TAS1R3 and TAS1R3 stimulated by calcium ions.
• IMP modulated sweet compound detection via the TAS1R3 subunit.

## 1 Introduction

Umami taste detection is mediated by the activation of the obligate heterodimeric G-protein-coupled receptor (GPCRs), named TAS1R1/TAS1R3, whereas the heterodimeric TAS1R2/TAS1R3 is specific to detecting sweet-tasting compounds (Belloir, Neiers, & Briand, 2017; Li et al., 2002; Nelson et al., 2001; Nelson et al., 2002). The TAS1R subunits are member of the Class C GPCR family. This family includes metabotropic glutamate receptors (mGluRs), γ-aminobutyric acid B-type receptors (GABA_B_Rs), extracellular calcium-sensing receptors (CaSRs), L-α-amino acid receptor GPRC6A and several orphan receptors, including some pheromone receptors (Brauner-Osborne, Wellendorph, & Jensen, 2007; Pin, Galvez, & Prézeau, 2003). Class C GPCRs share a common topology made of a large amino-terminal extracellular domain (ECD) that is composed of a Venus flytrap (VFT) domain and a cysteine-rich domain (CRD). The ECD is connected to the 7-helix transmembrane (7TM) domain by the CRD, except in the GABA_B_R subunits, which do not possess a CRD. Structural studies and site-directed mutagenesis have revealed that VFT is composed of two lobes separated by a large cleft forming the main orthosteric ligand-binding site (Kunishima et al., 2000; Muto, Tsuchiya, Morikawa, & Jingami, 2007). A combination of X-ray crystallography and cryo-electron microscopy has recently revealed the activation mechanism of the homodimeric human mGluR5 with a  sequential, multistep activation mechanism (Koehl et al., 2019; Krishna Kumar et al., 2024). Although the 3D structure of the human sweet and umami taste receptors is not known, the structure of the T1r2a-T1r3 VFT of the medaka fish, which responds to various L-amino acids, has been determined via crystallographic analysis (Ishida, Yasui, & Yamashita, 2024; Nuemket et al., 2017). Agonist binding to the VFTs leads to a compaction of the inter-subunit dimer interface, bringing the CRD into close proximity. Interactions between the CRDs and the second extracellular loops of the receptor lead to the reorientation of the 7TM, which initiates cascade signaling (Koehl et al., 2019). Several studies also suggest that class C GPCRs are activated by an agonist binding in the VFTs which stabilize the closed state and active the conformation of the VFT dimers leading to the movement of the CRDs and the conservation of the 7TMs promoting inter-subunit molecular contacts and stabilizing the active state of the receptor (Acher, Cabayé, Eshak, Goupil-Lamy, & Pin, 2022; Du et al., 2021; Krishna Kumar et al., 2024; Lebon, 2023; Lin et al., 2021; Nasrallah et al., 2021; Seven et al., 2021). Conversely, the binding of an antagonist maintains the VFTs in an open state and inactive the conformation of the dimer, preventing reorganization of the two subunits and inhibiting receptor activation (Nasrallah et al., 2021).

Despite sharing the TAS1R3 subunit, umami and sweet taste receptors detect and discriminate very different tasting compounds (Behrens, Meyerhof, Hellfritsch, & Hofmann, 2011; Belloir et al., 2017). TAS1R1/TAS1R3 responds to umami compounds such as L-amino acids, 5′-ribonucleotides and some peptides (Li et al., 2002; Nelson et al., 2001; Nelson et al., 2002; Toda et al., 2013; Xu et al., 2004; Zhao et al., 2003), whereas TAS1R2/TAS1R3 is involved in the detection of sugars, high-potency sweeteners, some D-amino acids, and sweet-tasting proteins (Jiang et al., 2004; Li et al., 2002; Masuda et al., 2018; Nelson et al., 2001; Nelson et al., 2002; Xu et al., 2004). The combination of site-directed mutagenesis, mouse-human chimeric receptors and cellular-based assays demonstrated that the TAS1R1- and TAS1R2-VFTs account for the interaction with the majority of umami and sweet-tasting compounds, respectively (Li et al., 2002; Maillet et al., 2015; Masuda et al., 2012; Toda et al., 2013; Xu et al., 2004; Zhang et al., 2008). However, other ligand binding sites have been shown on the receptors at the origin of synergy or receptor inhibition. TAS1R2-7TM was shown to interact with the sweet-tasting compounds perillartine and S819 (Cai et al., 2016; Servant, Tachdjian, Li, & Karanewsky, 2011; Zhang et al., 2008), and the sweet taste inhibitor amiloride (Zhao, Xu, Meng, & Liu, 2018). TAS1R1-7TM was shown to bind with the umami compound S807 (Zhang et al., 2008) whereas TAS1R3-7TM interacts with the sweeteners cyclamate and neohesperidine dihydrochalcone (NHDC) (Chéron et al., 2019; Fujiwara et al., 2012; Jiang et al., 2005; Winnig, Bufe, & Meyerhof, 2005; Xu et al., 2004). TAS1R3-7TM was also shown to interact with lactisole and clofibrate, two inhibitors of sweet and umami tastes (Galindo-Cuspinera & Breslin, 2006; Kochem & Breslin, 2017; Nakagita et al., 2019). Cyclamate which behaves as an agonist for the sweet taste receptor acts as an enhancer for the umami receptor (Zhang et al., 2008). We and others demonstrated that TAS1R3-VFT binds natural sugars (sucrose and glucose) and the chlorodeoxysugar sucralose (Maîtrepierre, Sigoillot, Pessot, & Briand, 2012; Nie, Vigues, Hobbs, Conn, & Munger, 2005). However, the role of TAS1R3-VFT and its contribution to heterodimeric sweet receptor function remain largely unknown (Zhang et al., 2022).

A unique feature of umami taste is its potentiation by 5′-ribonucleotides, such as inosine-5′-monophosphate (IMP) and guanosine-5′-monophosphate (GMP) (Li et al., 2002; Raliou et al., 2011; Servant & Frerot, 2022; Smith & Spector, 2014) which is the hallmark of umami taste. The molecular mechanism of this synergy involving a cooperative model has been elucidated. L-glutamate (L-Glu) binds in the hinge region of the TAS1R1-VFT, inducing the closure of the VFT, whereas IMP binds to an adjacent site, further stabilizing the closed conformation of the TAS1R1-VFT (Zhang et al., 2008).

Umami-sweet taste interactions have been highlighted by sensory analysis (Shim et al., 2015). For example, it has been shown that a moderate/high concentration of L-Glu suppresses sweet and bitter tastes (Kemp & Beauchamp, 1994), whereas 5′-ribonucleotides such as IMP enhance sweetness perception (Woskow, 1969). Using functional expression of the human TAS1R2/TAS1R3 sweet taste receptor, it has been demonstrated that umami compounds, including L-Glu, 5′-mononucleotides (IMP and GMP) and some glutamyl-dipeptides, inhibit the activation of the receptor by sweeteners (sucrose and acesulfame-K) that bind TAS1R2-VFT, but not by cyclamate, which interacts with the TAS1R3-7TM (Shim et al., 2015). While the roles of the individual subunits TAS1R1 and TAS1R2 in the interaction with umami and sweet compounds have been investigated (Li et al., 2002; Nelson et al., 2001; Nelson et al., 2002; Toda et al., 2013; Zhang et al., 2008; Zhang et al., 2010), the role of the TAS1R3 subunit in umami detection remains largely unknown.

This study aimed to understand the structural basis of umami stimulus recognition by the TAS1R1/TAS1R3 receptor using an in vitro functional assay. First, the two ligand-binding domains, TAS1R1- and TAS1R3-VFTs, were overexpressed in *E. coli* as previously described (Belloir, Savistchenko, et al., 2017; Laffitte, Belloir, Neiers, & Briand, 2022; Maîtrepierre et al., 2012; Maîtrepierre, Sigoillot, Le Pessot, & Briand, 2013). The folding and the structural integrity of proteins were confirmed by biophysical studies (western-blot, size-exclusion chromatography with multi-angle light scattering (SEC-MALS) and circular dichroism (CD) analyses). Fluorescent binding experiments were carried out to measure the affinity between TAS1R1- and TAS1R3-VFT domains, and L-Glu, L-Asp (L-aspartate), IMP and D-Glu (D-glutamate, as a negative control). The umami synergistic effect of IMP on the binding of L-Glu and L-Asp to TAS1R1- and TAS1R3-VFTs was also investigated. The activation of the homodimer TAS1R3/TAS1R3 and heterodimer TAS1R2/TAS1R3 by CaCl_2_ in the absence and presence of IMP or cyclamate was measured. Finally, the effect of umami compounds, including IMP, GMP and MSG (monosodium glutamate), on the activation of the TAS1R2/TAS1R3 sweet taste receptor by sucralose, neotame, perillartine, cyclamate and NHDC was investigated.

## 2 Material and methods

### 2.1 TAS1R-VFT expression

#### 2.1.1 Construction of the plasmid for *E. coli* expression and overexpression of recombinant TAS1R-VFT proteins

The cDNA sequences encoding TAS1R1-VFT (accession number NM_138697), TAS1R2-VFT (accession number NM_152232), or TAS1R3-VFT (accession number NM_152228) minus a putative signal sequence and the CRD were synthesized by DNA2.0 (Newark, CA, USA), optimized for expression in *E. coli* (Raliou et al., 2011), and subcloned into the *Nde*I and *Eco*RI restriction sites of the pET28a expression vector (Novagen, Sigma-Aldrich, Saint-Quentin-Fallavier, France). For each construct, the forward primer incorporated a *Nde*I sequence. The reverse primer incorporated an *Eco*RI sequence for cloning purposes, as well as a sequence coding for a His-tag for TAS1R1- and TAS1R2-VFT construction and a sequence coding for a Strep-tag II for TAS1R3-VFT. The final expression vectors pET28-TAS1R1-VFT and pET28-TAS1R2-VFT encoded a fusion protein comprising an N-terminal His_6_-tag that could be cleaved by thrombin, followed by TAS1R1-VFT (Phe21-Ser495) or TAS1R2-VFT (Ala22-Ser493) and a second His_6_-tag. pET28-TAS1R3-VFT encodes an N-terminal His_6_-tag followed by TAS1R3-VFT (Ala21-Ser497) and the Strep-tag II. The final constructs were checked by DNA sequencing (Genewiz, Leipzig, Germany) and were used for all subsequent studies with *E. coli*.

Overexpression of TAS1R-VFTs was carried out by inducing *E. coli* One Shot® strain BL21 Star™ (DE3) (Invitrogen, Life Technologies, Illkirch-Graffenstaden, France) grown on 2xYT medium (16 g/L tryptone, 10 g/L yeast extract and 5 g/L NaCl, pH 7.5) containing 45 μg/mL kanamycin at 37 °C to an optical density of 0.7 at 600 nm. Protein expression was induced for 3 h by the addition of 1 mM isopropyl-ß,D-thiogalactopyranoside (IPTG). The culture was harvested by centrifugation (4,500 x *g* for 15 min at 4 °C). The cell pellet was stored at −80 °C until further use. The overexpression of TAS1Rs-VFT was confirmed by SDS-PAGE and western blot analysis.

#### 2.1.2 Inclusion body (IB) purification

Purification of IB was carried out using detergent buffer, Triton buffer and a final urea wash protocol. Cells from 1 L of culture were resuspended in 20 mL of ice-cold lysis buffer (50 mM Tris-HCl pH 8.0, 50 mM NaCl, 0.2 % Tween 20, 0.5 % Triton X-100, 10 mM EDTA, 5 % glycerol, 1 mM PMSF) and disrupted by sonication (Vibra Cell, Sonics & Materials, Newtown, CT, USA) for 3 min. DNase I (10 μg/mL) and lysozyme (0.2 mg/mL) were added, and the cell suspension was incubated for 30 min at 600 rpm at 37 °C with a thermomixer. The lysed cell suspension was sonicated and centrifuged for 15 min at 10,000 x *g* at 4 °C. The supernatant was discarded, and the pellet, which contained IB, was washed with 10 mL of high salt buffer (50 mM Tris-HCl pH 8.0, 0.5 M NaCl, 0.5 % Triton X-100, 10 mM EDTA), sonicated for 2 min and centrifuged for 10 min at 10,000 x *g* at 4 °C. Then, two washes with urea buffer (50 mM Tris-HCl pH 8.0, 150 mM NaCl, 0.5 % Triton X-100, 10 mM EDTA, 4 M urea) were processed. Finally, the pellet was resuspended in Triton buffer (50 mM Tris-HCl pH 8.0, 150 mM NaCl, 0.5 % Triton X-100, 10 mM EDTA), aliquoted by 1 mL in a microtube, and centrifuged for 20 min at 10,000 x *g* at 4 °C. The supernatant was discarded, and the final pellet was dried and stored at −80 °C until use.

#### 2.1.3 Solubilization of IB and refolding of recombinant TAS1R-VFTs

IB were solubilized using high concentrations of urea as a chaotropic agent, in the presence of SDS and DDM (Anatrace, Maumee, OH, USA). The dried pellet was mixed with lysis buffer (50 mM Tris-HCl pH 8.0, 0.6 % SDS, 8 M urea, 25 mM DTT and 0.1 mM DDM) and disrupted by high-speed shaking with a tissue lyser (Qiagen, Hilden, Germany) for 3 min with carbon beads introduced into the microtube, followed by 2 h of agitation at 600 rpm at 50 °C with a thermomixer. The non-solubilized fraction was pelleted by centrifugation for 30 min at 24,000 x *g* at 20 °C. The solubilized fraction (20 mg/mL) was diluted drop by drop in 20 mL lysis buffer and incubated for 1 h at 50 °C in a water bath.

Initiation of refolding started with urea and SDS elimination by dialysis. The solubilized fraction was filtered through 0.45 μM filters and loaded into a dialysis tubing with a suitable molecular cut-off of 12–14 kDa. First, dialysis was produced to reduce the urea concentration (50 mM Tris-HCl pH 8.0, 0.6 % SDS, 1 M urea, 1 mM DTT and 0.1 mM DDM). After 2 h, the dialysis tubing was transferred to a buffer without urea and with a low level of SDS (50 mM Tris-HCl pH 8.0, 0.06 % SDS, 1 mM DTT and 0.1 mM DDM) for 2 h at room temperature. Then the dialyzed sample was filtered (0.22 μM) and transferred to an Erlenmeyer flask with a magnet stirrer. The DDM concentration was adjusted to reach 5 mM, followed by SDS precipitation by successive addition of NaCl (300 mM) and KCl (300 mM) under vigorous agitation. The white precipitate of SDS was eliminated by centrifugation at 48,400 x *g* for 45 min at 15 °C. The resulting supernatant was filtered (0.22 μM) and dialyzed two times under a dialysis tubing cut-off of 50 kDa against dialysis buffer (50 mM Tris-HCl pH 8, 150 mM NaCl, 0.1 mM DTT and 0.1 mM DDM) for 2 h and then overnight.

#### 2.1.4 Purification of TAS1R-VFTs

The last step of refolding was achieved by a nickel affinity purification step. The dialyzed sample was loaded onto a 1 mL His-trap HP column (GE Healthcare, Velizy-Villacoublay, France) in closed circuit for 24 h, allowing isolation of denatured protein. The fraction not retained by the IMAC step was concentrated to approximately 2 mL using a preparative scale concentrator (Vivaspin, Sartorius, Aubagne, France) with a molecular weight cut-off of 30 kDa. A final polishing step was performed by SEC to separate the monomeric receptor and higher-molecular-weight forms of the receptor. A Superdex 200 10/300GL column with an Akta Pure FPLC system (GE Healthcare) was used. The column was first equilibrated with at least 2 column volumes of wash buffer (50 mM Tris-HCl pH 8.0, 150 mM NaCl, and 0.1 mM DDM). The concentrated protein sample was loaded on the column and washed using wash buffer at 0.5 mL/min. Protein elution was monitored with UV absorbance at 280 nm, and fractions were automatically collected. Peak fractions were pooled and analyzed by western blotting. A calibration to correlate the elution volume with the molecular mass was performed with a standard mixture.

### 2.2 TAS1R-VFT characterization

#### 2.2.1 Western blot analysis

TAS1R-VFTs were detected and their purity assessed using SDS-PAGE (Bio-Rad, Hercules, CA, USA) combined with Coomassie staining and western blot analysis. For blotting, the gel-resolved samples were transferred to PVDF membranes, blocked in skim milk (5 % *w*/*v* non-fat dried milk in TBST) for 1 h, and incubated with a His primary antibody (1: 2000 in TBST) overnight at 4 °C. The His-tagged proteins were then detected with a goat anti-mouse HRP-conjugated secondary antibody (Bio-Rad) (1:25,000 in TBST, 1 h, room temperature) and visualized using Clarity Western ECL substrate (Bio-Rad). All images were captured using the ChemiDoc system and Image Lab software (version 6.1.0 build 7 – standard edition, Bio-Rad) to analyze band migrations, intensities and purity.

#### 2.2.2 SEC-MALS analysis

The oligomeric state of the purified TAS1R-VFT proteins was analyzed by SEC-MALS using Jasco PU-2080 Plus HPLC system (Jasco, Cremella, Italy), consisting of a pump, a vacuum degasser and an autosampler, and a Silica Gel KW803 column (Shodex, Munich, Germany). Detection was performed using a triple-angle light scattering detector (miniDAWN™ TREOS, Wyatt Technology, Santa Barbara, CA, USA), a refractometer (Ri-4030 Refractive Index Detector, Jasco) and a UV detector (UV-4070 UV/Vis Detector, Jasco) at 280 nm. Molecular weight determination was performed using ASTRA software (version 6.1.5.22, Wyatt Technology) using a dn/dc value of 0.185 mL/g for analysis. The SEC-MALS system was calibrated with bovine serum albumin prior to TAS1R-VFT proteins. The gel filtration-purified fraction was loaded onto a pre-equilibrated column with wash buffer as previously described. An aliquot of 100 μL of purified TAS1R-VFT fractions at a concentration of 1.2 mg/mL was injected with a flow rate of 0.5 mL/min.

#### 2.2.3 CD analysis

Far-UV CD spectra were measured over 180–260 nm with a CD spectropolarimeter (Jasco J-815) equipped with a Peltier temperature control. The fractions were concentrated to 0.4 and 0.2 mg/mL for TAS1R1- and TAS1R3-VFTs, respectively. Measurements were made at 20 °C in a quartz cuvette with a path length of 0.01 cm, a step size of 0.5 nm, a scan speed of 50 nm/min and a response time of 1 s. The data were averaged over 10 accumulated scans. The spectra were smoothed using the Stavitzky-Golay convolution filter with a span of 5. They were corrected for buffer contribution and converted to mean residue ellipticity in deg.·cm^2^·dmol^−1^. The secondary structure proportions were estimated using the deconvolution K2D algorithm on the DichroWeb program (http://dichroweb.cryst.bbk.ac.uk/html/home.shtml) (Institute of Structural and Molecular Biology, Birkbeck College, University of London, London, UK; accessed on March 2017).

### 2.3 TAS1R-VFT ligand binding analysis using intrinsic fluorescence

The intrinsic fluorescence of TAS1R-VFTs was measured using a Cary Eclipse spectrofluorometer (Varian Instruments, Palo Alto, CA, USA) equipped with a Peltier temperature control unit. Sample proteins were excited at 295 nm, and emission spectra were recorded between 300 and 400 nm, with a 5-nm slit width for emission and excitation. For titration experiments, fluorescence spectra were recorded using 0.25 μM protein solution in 50 mM Tris-HCl pH 8.0, 150 mM NaCl, and 0.1 mM DDM. Ligand solutions were freshly prepared in the same buffer. Successive aliquots of potential ligands (including L-Glu, L-Asp, IMP and D-Glu as a negative control) were added to 400 μL of TAS1R-VFT solution. The temperature was kept constant at 20 °C. Fluorescence measurements were corrected for dilution, bleaching and nonspecific buffer quenching. *K*_d_ values and standard errors were calculated from a plot of the ratio between the fluorescence intensity variation and the maximum of fluorescence intensity variation versus concentration of total ligand, obtained with a standard non-linear regression method and using a one-site saturation ligand binding equation within SigmaPlot software (version 15.0, Grafiti, Palo Alto, CA, USA). To measure the synergism between L-amino acids (L-Glu and L-Asp) and IMP, the fluorescence intensities of TAS1R-VFTs were normalized to 0 % in the presence of 0.3 μM IMP alone and 100 % at the highest concentrations of L-amino acids in the absence of IMP. The *K*_d_ values of ligand-receptor interactions were determined using the equation ΔF/ΔF_max_ = B_max_ [L]/(*K*_d_ + [L]), where *K*_d_ is the apparent dissociation constant, ΔF is the difference between the fluorescence intensity at a given concentration of ligand and the fluorescence intensity in the absence of ligand, ΔF_max_ is the difference at infinite ligand concentration [L], B_max_ is the maximum fluorescence signal, and [L] corresponds to the concentration of ligands. The reported *K*_d_ values are the average of six measurements performed on at least three independently refolded protein samples. All compounds were obtained from Sigma-Aldrich. Statistical analyses: Dunnett's test (*p* ≤ 0.05) was used to determine the statistical significance between data means.

### 2.4 Construction of the expression plasmid for functional expression of TAS1R proteins in HEK293 cells stably expressing Gα16gust44

The chimeric Gα16gust44 protein was generated by replacing the C-terminus of Gα16 with the C-terminal 44 residues of Gα-gustducin (Raliou et al., 2011). The synthetic cDNA optimized for human TAS1R2 and TAS1R3 expression was cloned into the pcDNA6-myc-HisA and pcDNA4-myc-HisA vectors, generating the pcDNA6-TAS1R2 and pcDNA4-TAS1R3 plasmids, respectively (Genewiz).

### 2.5 Cell culture and transfection

Human embryonic kidney (HEK) 293T cells were cultured in DMEM supplemented with 10 % dialyzed FBS, 1 % P/S at 37 °C and 7.3 % CO_2_ in a humidified atmosphere. The stable HEK293T-Gα16gust44 cells were constructed by stable transfection of pcDNA3.1/Hygro-Gα16gust44 plasmid into HEK293T cells using Lipofectamine 2000 (Invitrogen). After 48 h, the cells were selected with 400 μg/mL hygromycin for 2–3 weeks. Thirty individual hygromycin-resistant colonies were expanded and screened for functional expression after transient co-transfection with pcDNA6-TAS1R2 and pcDNA4-TAS1R3 and measurement of calcium responses to sucralose. In the same way, clone selection was done under zeocin selection (250 μg/mL) to generate a stable HEK293T-Gα16gust44-TAS1R3 cell line for further calcium mobilization assays.

### 2.6 Calcium mobilization assays

Forty-eight hours before the assay, HEK293T-Gα16gust44 or HEK293T-Gα16gust44-TAS1R3 cells were plated (4 × 10^4^ cells/well) on poly-d-lysine-coated 96-well plates. Twenty-four hours later, cells were transiently transfected with pcDNA6-TAS1R2 and pcDNA4-TAS1R3 with Lipofectamine 2000 according to the manufacturer's instructions. As a negative control, HEK293T cells were mock-transfected with the empty expression vector. Twenty-four hours after transfection, the cells were loaded with the calcium indicator Fluo-4 AM (2.5 μM, Molecular Probes, Invitrogen) dissolved in pluronic acid (0.025 %, *w*/*v*) in the presence of 2.5 mM probenecid for 1 h at 37 °C. After washing with C1 buffer (130 mM NaCl, 5 mM KCl, 10 mM Hepes pH 7.4, 2 mM CaCl_2_), the 96-well plates containing the cells were transfected into an automated fluorimetric FlexStation® 3 Multi-Mode Microplate Reader (SoftMax Pro 5.4.6; Molecular Devices, San Jose, CA, USA) and subjected to stimuli. The fluorescence intensity was measured for 90 s after the addition of the compounds. A range of concentrations from 1 to 1,000 μM of sucralose and 0.3 to 30 μM of neotame were tested on the activation of TAS1R2/TAS1R3 with or without IMP (0.1 or 10 mM), GMP (0.1 mM or 10 mM) and MSG (10 mM) alone. Moreover, another range of concentrations from 0.1 to 300 μM of perillartine, 10 to 10,000 μM of cyclamate and 1 to 300 μM of NHDC were tested on the same receptor with or without IMP (0.1 or 10 mM). Then, a range of concentrations from 1 to 100 mM of CaCl_2_ were tested on TAS1R3 and TAS1R2/TAS1R3 with and without IMP (10 mM) or cyclamate (10 mM). All concentration-receptor combinations were measured in triplicate, and each experiment was repeated at least four times. The Ca^2+^ changes are expressed as fractional changes in fluorescence light intensity: ΔF/F_0_ = (F—F_0_)/F_0_, where F is the fluorescence light intensity at each point and F_0_ is the value of emitted fluorescent light before the stimulus application. For the calculation of dose-response relationships, the changes in fluorescence upon stimulus application were averaged, mock-subtracted and baseline-corrected. The resulting dose-response data were fitted using a four-parameter logistic equation. The half-maximal effective concentrations (EC_50_ values) were calculated using SigmaPlot software.

## 3 Results and discussion

### 3.1 Expression and biophysical characterization of TAS1R1- and TAS1R3-VFTs

In order to assess the role of each subunit in umami taste receptor function, we produced recombinant hTAS1R1- and hTAS1R3-VFTs in bacteria as previously reported (Maîtrepierre et al., 2013). This strategy was previously used to express functionally active cat umami TAS1R1-VFT (Belloir, Savistchenko, et al., 2017) and human TAS1R2- and TAS1R3-VFTs (Laffitte et al., 2022; Maîtrepierre et al., 2012). The cDNAs coding for TAS1R1- and TAS1R3-VFTs were cloned into a bacterial expression vector and overexpressed in *E. coli* BL21(DE3) cells (Fig. S1a and b). SDS-PAGE and western blot analysis showed a band migrating at around 50 kDa (Fig. S1c and d), revealing that TAS1R1- and TAS1R3-VFTs were highly expressed after IPTG induction. IB were isolated, washed and approximately 185 and 45 mg were obtained from 1 L of bacterial culture for TAS1R1- and TAS1R3-VFTs, respectively. TAS1R1- and TAS1R3-VFT proteins were refolded using the protocol previously described (Belloir, Savistchenko, et al., 2017) with slight modifications (Fig. S2). Isolation of the monomeric refolded proteins was performed using SEC. As shown in Fig. S3, TAS1R1- and TAS1R3-VFTs eluted at 14.5 mL as a single well-defined peak. The calibration of the column with molecular mass markers (Fig. S4) indicated that proteins had apparent molecular masses of 56 and 60 kDa for TAS1R1- and TAS1R3-VFTs, respectively, in agreement with monomeric structures of both proteins. SDS-PAGE and western blot analysis using anti-His-tag antibodies of the eluted fractions confirmed that TAS1R1- and TAS1R3-VFTs were the main proteins present in the samples (Fig. S5). To confirm the correct folding of TAS1R1- and TAS1R3-VFTs, we performed CD spectroscopy. The CD spectra displayed α-helix-dominant secondary structures with a positive peak centered at 193 nm and two negative peaks, at wavelengths of 208 and 222 nm, for both TAS1R-VFTs (Fig. S6). The deconvolution of the CD spectra revealed that TAS1R1- and TAS1R3-VFTs had a high content of helical secondary structures, in accordance with those measured in human and mouse TAS1R2- and TAS1R3-VFTs (Maîtrepierre et al., 2012; Nie et al., 2005).

The oligomeric state of TAS1R3- and TAS1R1-VFTs was further analyzed using SEC-MALS. SEC-MALS analysis (Fig. S7) showed that TAS1R1- and TAS1R3-VFTs eluted as monodisperse peaks with measured masses of 55 and 56 kDa, respectively. These data confirmed the monomeric structure of TAS1R1- and TAS1R3-VFTs. Then, we recorded the intrinsic tryptophan fluorescence spectra to probe the refolded state of TAS1R1- and TAS1R3-VFTs in the presence and absence of 6 M guanidine hydrochloride (GuCl) as a chaotropic agent. Denaturation of TAS1R1- and TAS1R3-VFTs with GuCl caused a red shift of the emission maximum from 335 to 350 nm and a 20 % decrease in fluorescence intensity (Fig. S8), indicating that both proteins were folded prior to denaturation.

Altogether, our results showed that both TAS1R1- and TAS1R3-VFT can be expressed in *E. coli* as soluble and correctly refolded proteins. Therefore, these proteins were suitable for subsequent functional analyses.

### 3.2 Interaction of TAS1R-VFTs with L-Glu, L-asp, IMP and D-Glu in the absence or presence of IMP

To determine the distinct contributions of TAS1R1- and TAS1R3-VFTs to the detection of umami stimuli, we measured their interaction with four compounds, including L-Glu, L-Asp, IMP and D-Glu. The interaction was also measured for L-Glu and L-Asp in presence of 0.3 μM IMP. Therefore, we determined the concentration-response relationships for the intrinsic tryptophan fluorescence of the TAS1R-VFT proteins upon titration with these compounds. This fluorescence technique has been applied successfully to measure protein-ligand binding interactions with the VFTs of class C GPCRs, including TAS1R receptors (Belloir, Brulé, Tornier, Neiers, & Briand, 2021; Belloir, Savistchenko, et al., 2017; Huang et al., 2009; Maîtrepierre et al., 2012; Nie et al., 2005; Nie et al., 2006; Suzuki, Moriyoshi, Tsuchiya, & Jingami, 2004).

**Fig. 1:**
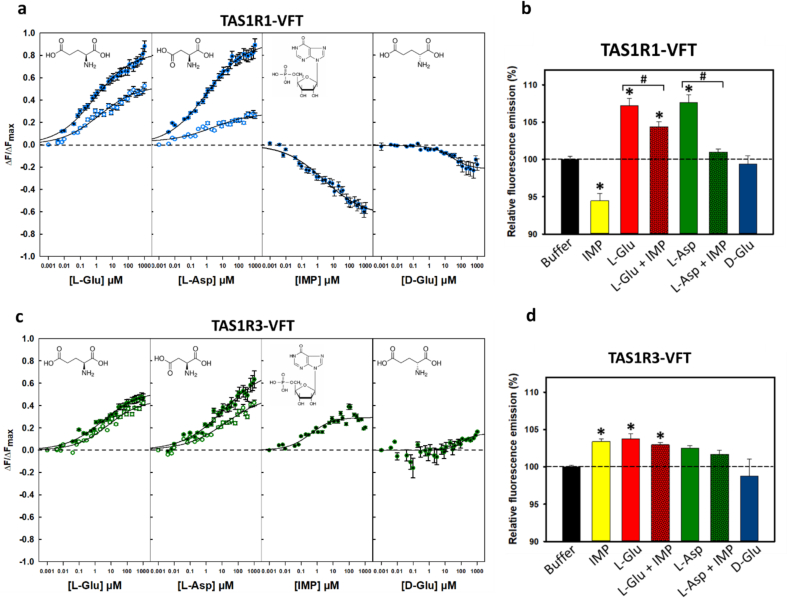
TAS1R1 and TAS1R3-VFTs bind L-amino acids and IMP. Titration curves of TAS1R1-VFT (a) and TAS1R3-VFT (c) with IMP alone and umami L-amino acids in the presence (open circles) or absence of 0.3 μM IMP (filled circles). Circles show experimental data, while the solid lines are the computed binding curves; excitation and emission wavelengths were 295 and 340 nm, respectively; TAS1R-VFT concentration was 0.5 μM. Data points correspond to mean ± SEM of more than ten independent replicates of at least six independently refolded protein samples. Data were fitted with sigmoid dose-response curves using SigmaPlot software. The chemical structure of each compound is shown. Intrinsic tryptophan fluorescence of TAS1R1-VFT (b) and TAS1R3-VFT (d) alone was defined as 100 % in the absence of tastant. *, # Significantly different from TAS1R-VFT before addition of ligands (one-way ANOVA followed by Dunnett's, *p* ≤ 0.05). VFT: Venus flytrap domain; L-Glu: L-glutamate; L-Asp: L-aspartate; IMP: inosine-5′-monophosphate; D-Glu: D-glutamate.

As expected, we found that L-Glu and L-Asp alone induced conformational changes in TAS1R1-VFT leading to a saturable intrinsic fluorescence enhancement (Fig. 1a). L-Glu and L-Asp bound TAS1R1-VFT with distinct *K*_d_ values of 0.11 ± 0.03 and 0.21 ± 0.06 μM, respectively (Table 1). This affinities in the micromolar range were in agreement with their respective umami potencies evaluated by sensory analysis (Kawai, Okiyama, & Ueda, 2002; Yamaguchi, Yoshikawa, Ikeda, & Ninomiya, 1971). These *K*_d_ values were lower than the predicted values deduced from the sensory analysis or cellular-based assays (Li et al., 2002; Nelson et al., 2002; Toda et al., 2013; Zhang et al., 2008), as previously observed for cat TAS1R1-VFT, and human and mouse TAS1R2-VFTs (Belloir, Savistchenko, et al., 2017; Maîtrepierre et al., 2012; McGrane et al., 2023; Nie et al., 2005). The addition of IMP in the absence of L-amino acids resulted in a saturable decrease of TAS1R1-VFT fluorescence leading to a high affinity (*K*_d_ = 0.06 ± 0.02 μM; Fig. 1a, Table 1). This decrease of fluorescence suggests a binding-dependent conformational change, which is different from that induced by L-Glu and L-Asp binding. Since the molecular mechanism of IMP synergy involves TAS1R1-VFT, we investigated the synergy between IMP and the two umami L-amino acids, L-Glu and L-Asp. The presence of IMP (0.3 μM, final concentration) decreased the maximal amplitude of TAS1R1-VFT fluorescence induced by L-Glu and L-Asp (Fig. 1b). L-Glu exhibited similar *K*_d_ values in the absence or presence of IMP whereas the addition of IMP highly increased the *K*_d_ value of L-Asp showing a lower affinity for this amino acid and TAS1R1-VFT (Table 1). In contrast with previous observations for cat TAS1R1-VFT (Belloir, Savistchenko, et al., 2017), we did not observe a synergistic effect of IMP on the binding of these amino acids to TAS1R1-VFT.

**Table 1:** Binding properties of TAS1R1- and TAS1R3-VFT proteins assessed by intrinsic tryptophan fluorescence in the absence or presence of 0.3 μM IMP (only for L-Glu and L-Asp). The dissociation constants (*K*_d_) values are reported as the mean ± SEM.

Ligand	*K*_d_ TAS1R1-VFT (μM)	*K*_d_ TAS1R3-VFT (μM)
L-Glu	0.11 ± 0.03	0.75 ± 0.20
L-Glu + IMP	0.16 ± 0.04	1.37 ± 0.30
L-Asp	0.21 ± 0.06	1.18 ± 0.33
L-Asp + IMP	1.49 ± 0.64	1.41 ± 0.46
IMP	0.06 ± 0.02	0.22 ± 0.07
D-Glu	nb	nb

VFT: Venus flytrap; L-Glu: L-glutamate; IMP: inosine-5′-monophosphate; L-Asp: L-aspartate; D-Glu: D-glutamate; nb: no binding.

We then carried out the same experiment using TAS1R3-VFT. Interestingly, we observed that TAS1R3-VFT exhibited a dose-dependent increase in intrinsic fluorescence by L-Glu and L-Asp leading to *K*_d_ values of 0.75 ± 0.20 and 1.18 ± 0.33 μM, respectively (Fig. 1c, Table 1). However, the affinities were lower compared to those measured on TAS1R1-VFT (Table 1). Surprisingly, we found that TAS1R3-VFT exhibited a dose-dependent increase in fluorescence by IMP alone, leading to *K*_d_ value of 0.22 ± 0.07 μM that was higher than those of TAS1R1-VFT. The increase in TAS1R3-VFT fluorescence upon IMP binding in the absence of umami L-amino acids suggests a different ligand-dependent conformational change compared to that observed with TAS1R1-VFT. We also investigated the effect of IMP (0.3 μM, final concentration) on the ability of TAS1R3-VFT to bind these L-amino acids. In the presence of IMP, L-Glu and L-Asp bound TAS1R3-VFT with the same relative order of *K*_d_ values although the affinity of L-Glu was higher in the absence of IMP (Fig. 1d, Table 1). The addition of D-Glu, as a negative control, had no significant impact on the fluorescence intensity of TAS1R1- and TAS1R3-VFTs (Fig. 1a and c). This result was consistent since D-Glu was not perceived as umami by humans (Yamaguchi, 1991) and did not activate the human TAS1R1/TAS1R3 receptor (Li et al., 2002).

Taking together, our results demonstrate that both TAS1R1- and TAS1R3-VFTs are able to bind umami compounds at physiologically relevant concentrations. Interestingly, we found that TAS1R3-VFT binds umami stimuli and may contribute to umami receptor activation.

### 3.3 Effect of IMP and cyclamate on the activation of TAS1R3/TAS1R3 and TAS1R2/TAS1R3 stimulated by CaCl_2_ using cellular assay

**Fig. 2:**
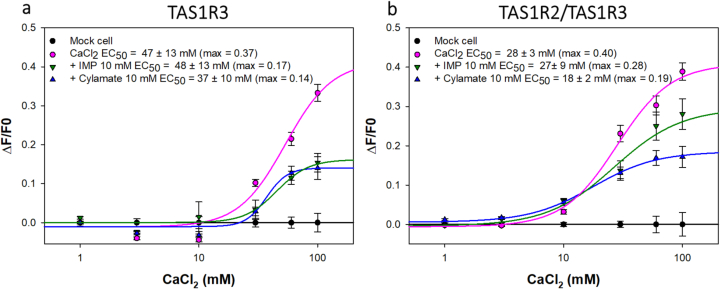
Dose-dependent responses of TAS1R3 alone (a) and TAS1R2/TAS1R3 (b) stimulated by CaCl_2_ in the absence and presence of IMP or cyclamate_._ TAS1R3 was stably expressed in HEK293T-Gα16gust44 cells without TAS1R2 (a) or with TAS1R2 (b) transiently transfected and stimulated with CaCl_2_ alone (pink line) or in the presence of 10 mM IMP (green line) or 10 mM cyclamate (blue line). Changes in fluorescence after CaCl_2_ stimulation, were monitored and plotted on the y-axes. (ΔF/F_0_) was calculated and fitted with sigmoidal regression with SigmaPlot software. The results are shown as the mean ± SEM of at least three independent experiments. IMP: inosine-5′-monophosphate. (For interpretation of the references to colour in this figure legend, the reader is referred to the web version of this article.)

It has been shown that the TAS1R3 subunit, expressed alone, probably acting as a TAS1R3/TAS1R3 homodimer, did not respond to umami stimuli or to sweeteners but was activated by calcium ions whose activation was reduced in the presence of lactisole (Tordoff, Alarcón, Valmeki, & Jiang, 2012). Since we found that IMP interacts with TAS1R3-VFT, we investigated the effect of IMP, and cyclamate, which is known to bind TAS1R3-7TM (Jiang et al., 2005), on the activation of the TAS1R3 homodimer and the TAS1R2/TAS1R3 sweet taste receptor heterodimer both stimulated by calcium ions. The concentration-response curves, the maximal amplitude and the EC_50_ values are presented in Fig. 2. We found that cells co-expressing TAS1R2 and TAS1R3 subunits responded to calcium in a dose-dependent manner, leading to a lower EC_50_ value (28 ± 3 mM) than that observed for the TAS1R3 subunit alone (47 ± 13 mM), which was consistent with the previous study (Tordoff et al., 2012). In the presence of 10 mM IMP and 10 mM cyclamate, the amplitude of the maximum calcium-induced response of TAS1R3 was reduced to approximately 54 and 62 %, respectively, but led to comparable EC_50_ values (Fig. 2a). Concerning TAS1R2/TAS1R3 stimulated by calcium ions, the addition of 10 mM IMP and 10 mM cyclamate did not modify the EC_50_ values but the signal amplitudes were decreased up to 30 % and 52 %, respectively (Fig. 2b). Our data suggest that IMP and cyclamate bind to TAS1R2/TAS1R3 and are able to modulate the sweet taste receptor acting via the TAS1R3 subunit (non-competitive antagonists).

### 3.4 Effect of IMP, GMP and MSG on the activation of TAS1R2/TAS1R3 stimulated by five different sweeteners

Since TAS1R3 is the common subunit shared by the sweet and umami taste receptors, and is a binding site for some umami compounds, we hypothesize that some umami-tasting compounds may potentiate the sweetener-induced response of the TAS1R2/TAS1R3 receptor. To test this hypothesis, we performed functional expression of the full-length heterodimeric sweet taste TAS1R2/TAS1R3 receptor using HEK293-derived cells. We investigated the modulation of TAS1R2/TAS1R3 by umami compounds (IMP, GMP and MSG) stimulated by fives sweeteners (sucralose, neotame, perillartine, cyclamate and NHDC). We also tested the modulation effect of GMP and MSG for the activation of TAS1R2/TAS1R3 by sucralose and neotame.

**Fig. 3:**
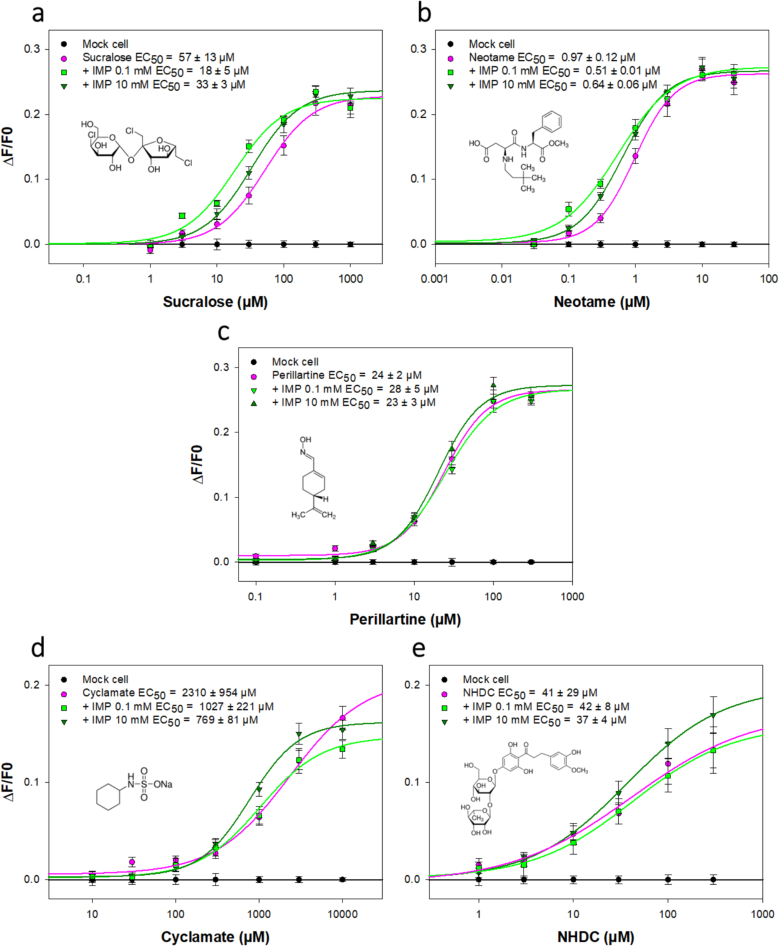
Dose-dependent responses of TAS1R2/TAS1R3 to various sweet compounds in the absence and presence of IMP (a-e). HEK293T-Gα16gust44 cells expressing TAS1R2/TAS1R3 were challenged with sweet compounds alone (pink line) or the sweet compounds in the presence of 0.1 mM IMP (light green line) or 10 mM IMP (dark green line). The cells were assayed for intracellular calcium increase using Fluo-4 as calcium probe. The data were fitted with sigmoid dose-response curves, and the EC_50_ values were calculated using SigmaPlot software. The results are shown as the mean ± SEM of at least three independent experiments. IMP: inosine-5′-monophosphate; NHDC: neohesperidine dihydrochalcone. (For interpretation of the references to colour in this figure legend, the reader is referred to the web version of this article.)

**Fig. 4:**
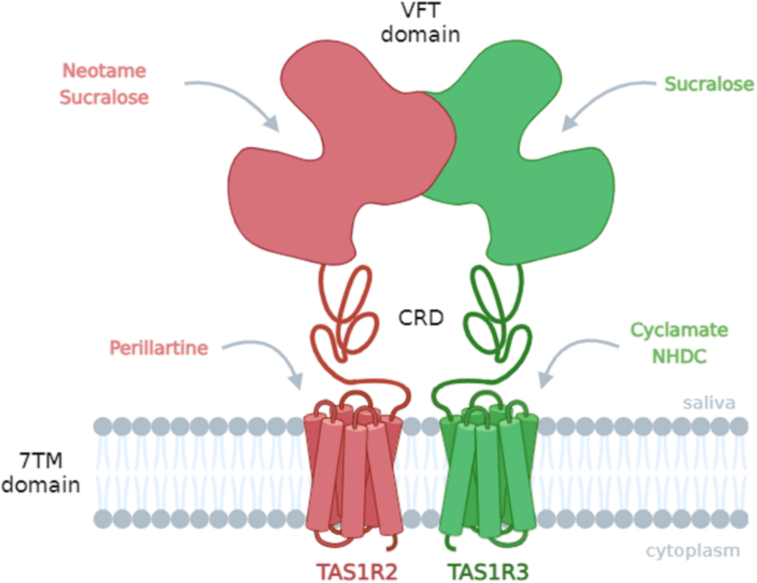
Schematic summary of the different sweet taste receptor (TAS1R2/TAS1R3) known binding sites of sucralose, neotame, perillartine, cyclamate and NHDC (Cai et al., 2016; Jiang et al., 2005; Maîtrepierre et al., 2012; Nie et al., 2005; Servant et al., 2011; Winnig et al., 2007; Xu et al., 2004). NHDC: neohesperidine dihydrochalcone; VFT: Venus flytrap domain; CRD: cysteine-rich domain; 7TM: 7-helix transmembrane.

The concentration response curves and the EC_50_ values of the five sweeteners in the presence of different concentrations of IMP (0, 0.1 and 10 mM) are presented in Fig. 3. The EC_50_ values of sucralose, neotame, perillartine, cyclamate and NHDC alone were consistent with the literature (Belloir et al., 2021; Choi et al., 2021; Servant et al., 2010; Xu et al., 2004). We found that the addition of IMP induced a decrease of the EC_50_ values of sucralose, neotame and cyclamate showing a moderate synergy. Intriguingly, the strongest synergistic effect was observed for the smallest IMP concentration for sucralose and neotame. However, at these concentrations, we observed that IMP did not modify the EC_50_ values of perillartine and NHDC. The TAS1R2/TAS1R3 receptor binding sites for these five sweeteners are well known. It has been demonstrated that neotame binds TAS1R2-VFT (Xu et al., 2004), while sucralose interacts with the VFTs of both TAS1R2 and TAS1R3 subunits (Maîtrepierre et al., 2012; Nie et al., 2005). It has been shown that perillartine binds 7TM of TAS1R2 (Cai et al., 2016) whereas cyclamate and NHDC are known to interact with TAS1R3-7TM (Jiang et al., 2005; Winnig, Bufe, Kratochwil, Slack, & Meyerhof, 2007). A summary of the taste receptor binding sites of the tested sweeteners is presented in Fig. 4. The synergistic effects observed for sucralose/IMP and neotame/IMP blends could be explained by the activation of at least two different sites of the sweet taste receptor in the VFT (Cui et al., 2006; Servant et al., 2011). Indeed, TAS1R2-VFT binds sucralose and neotame whereas sucralose and IMP interact with TAS1R3-VFT. Interestingly, the synergistic effect of IMP was higher for sucralose than neotame (decrease in EC_50_ values of 3-fold and 1.2-fold, respectively) which could be explained by the activation of both TAS1R2- and TAS1R3-VFTs by sucralose. Although IMP does not share binding sites with perillartine and NHDC, no synergy was detected between these compounds. The activation of TAS1R3-7TM and TAS1R3-VFT by cyclamate and IMP, respectively, could explain the synergetic effect observed among this blend which, surprisingly, was not observed for the NHDC/IMP blend. Our results are consistent with a sensory analysis that highlighted the enhancement of the sweet perception by IMP (Woskow, 1969) and suggest that TAS1R3 subunit is involved in the enhancing activity of IMP.

**Fig. 5:**
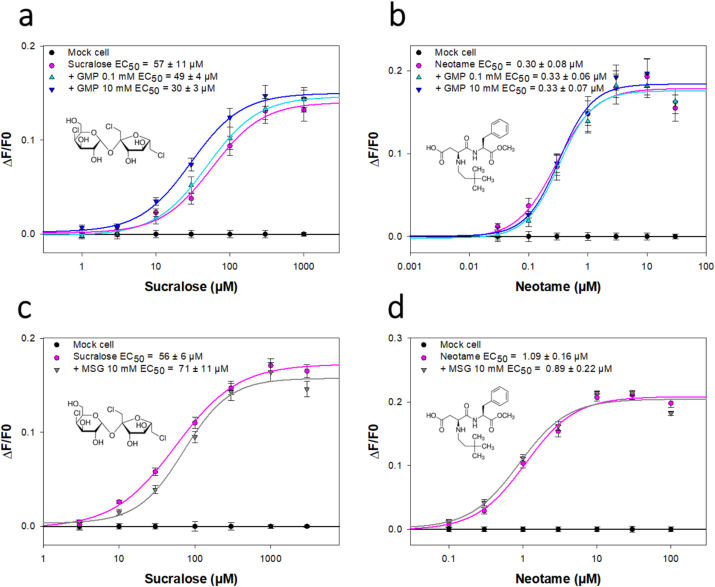
Dose-dependent responses of TAS1R2/TAS1R3 to sucralose (a and c) and neotame (b and d) in the absence and presence of GMP (a and b) or MSG (c and d). HEK293T-Gα16gust44 cells expressing TAS1R2/TAS1R3 were challenged with sweet compounds alone (pink line) or the sweet compounds in the presence of 0.1 mM GMP (light blue curve) or 10 mM GMP (dark blue line) or 10 mM MSG (grey curve). The cells were assayed for intracellular calcium increase using Fluo-4. The data were fitted with sigmoid dose-response curves, and the EC_50_ values were calculated using SigmaPlot software. The results are shown as the mean ± SEM of at least three independent experiments. GMP: guanosine-5′-monophosphate; MSG: monosodium glutamate. (For interpretation of the references to colour in this figure legend, the reader is referred to the web version of this article.)

The concentration response curves and the EC_50_ values of sucralose and neotame in the presence of different concentrations of GMP (0, 0.1 and 10 mM) and MSG (0 and 10 mM) are presented in Fig. 5. GMP did not enhance the sweet taste receptor activity for neotame, whereas stimulation in the presence of 10 mM GMP led to a decrease in the EC_50_ value for sucralose. This observation was consistent with a previous in vitro study that showed no effect of 0.3–3.0 mM GMP on the TAS1R2/TAS1R3 activation by sucrose (0–150 mM) (Shim et al., 2015). The addition of MSG did not modify significantly the EC_50_ values of sucralose and neotame. Our result are not consistent with that of a previous study which demonstrated the inhibitory effect of MSG on the activation of the sweet taste receptor by sweeteners that bind the ECD of TAS1R2, such as sucrose and acesulfame K (Shim et al., 2015). As sucralose and neotame interact with the TAS1R2-VFT, we should have observed an increase in the EC_50_ values for these sweeteners in the presence of MSG. However, the inhibitory effect was observed for sucralose concentrations of 100–150 mM in the presence of 10–50 mM MSG (Shim et al., 2015) suggesting that it would be interesting to study the inhibition of the sweet taste receptor with higher concentration of MSG.

The main contribution of TAS1R1 subunit regarding umami ligand interactions and IMP synergism acting through TAS1R1-VFT has been demonstrated (Li et al., 2002; Xu et al., 2004; Zhang et al., 2008). A previous study has shown that the addition of IMP (2.5 mM) did not modify the activation of TAS1R2/TAS1R3 taste receptor to sweet-taste stimuli such as sucrose (100 mM), aspartame (1.5 mM) and saccharin (0.4 mM) (Li et al., 2002). This was probably because the authors only tested high sweetener concentration near EC_100_ value instead of performing a dose-response curve with a lowest concentration of IMP. Interestingly, the sweet-umami chimeric receptor, which combines TAS1R2-ECD linked to TAS1R1-7TM with the TAS1R3 subunit, responded to sucrose and aspartame but not cyclamate (Zhang et al., 2008). However, these authors showed that cyclamate, instead of activating the sweet-umami chimeric receptor TAS1R2‐–1/TAS1R3, acted as an enhancer for sucralose and aspartame because of differences in the energy barrier of activation of these chimeric receptors. Surprisingly, these authors showed that IMP cannot enhance the activity of sucralose or aspartame on TAS1R2‐1/TAS1R3. These results differ from our observation that IMP could enhance the activity of sucralose and neotame on the wild-type sweet receptor (Zhang et al., 2008).

Few studies have been interested in the interaction between umami compounds and sweet taste receptors at the molecular level. For example, the investigation of the interaction of MSG and glutamyl dipeptides on sweet taste receptors showed that umami compounds might inhibit agonist binding to TAS1R2 in an allosteric manner (Shim et al., 2015). Our data suggest that a mechanism of inter-subunit and intra-subunit rearrangement could account for these results. We propose a hypothesis that IMP induces different conformational changes. First, IMP binding to TAS1R3-VFT induces conformation changes in the VFT domain, and the reorientation of the VFT domain can in turn lead to intra-subunit movement between TAS1R3-VFT and TAS1R3-7TM due to the rigidity of the CRD (Huang et al., 2011; Koehl et al., 2019; Muto et al., 2007). Thus, IMP could act as a cyclamate enhancer through rearrangement between TAS1R3- and TAS1R2-7TM when the sweet-tasting compound binds the VFT. The coupling cannot occur when 7TM is replaced by TAS1R1-7TM instead of TAS1R2-7TM in the TAS1R2 subunit, maybe because of the energy barrier, as suggested before (Zhang et al., 2008), or because TAS1R2-7TM still plays a role in the activation process. Second, IMP binding to TAS1R3-VFT can create inter-subunit rearrangement by allosteric interaction between the two VFT domains. Thus, displacement of the VFT domain equilibrium enhance TAS1R2/TAS1R3 activation by sweet-tasting ligands.

Moreover, these arguments are supported by many studies conducted on CaSR, mGlu and GABA_B_ receptors that have elucidated the mechanism of the functional interaction between each subunit and G proteins (Jacobsen, Gether, & Bräuner-Osborne, 2017; Koehl et al., 2019; Liu et al., 2017; Møller, Moreno-Delgado, Pin, & Kniazeff, 2017; Pin, Kniazeff, Prézeau, Liu, & Rondard, 2019). It has been shown for the mGlu dimeric receptor that glutamate binding to one VFT domain leads to receptor activation and facilitates the occupancy of the other VFT, inducing the full activity of the mGlu receptor (Kniazeff et al., 2004; Suzuki et al., 2004). Similarly, the heterodimeric cooperative phenomenon has been observed for GABA_B_ receptor, whose agonist affinity towards GABA_B1_ is enhanced by its interaction with the VFT of GABA_B2_ (Geng, Bush, Mosyak, Wang, & Fan, 2013). This mechanism is also confirmed for the CaSR dimer, showing that a single allosteric site is sufficient for obtaining positive allosteric modulator (PAM) activity (Jacobsen et al., 2017). The TAS1Rs assemble to form heterodimers, TAS1R1/TAS1R3 for umami receptor and TAS1R2/TAS1R3 for sweet taste receptor (Li et al., 2002; Xu et al., 2004), and TAS1R3 in homomeric form is able to respond to high concentrations of sucrose (Nelson et al., 2001; Zhao et al., 2003). Several studies indicate that the dimeric structure is essential for the conformational transitions to G protein activation (El Moustaine et al., 2012; Pin & Bettler, 2016). It is not yet clear which 7TM between TAS1R1, T1S1R2 and TAS1R3 is responsible for coupling to G proteins in sweet and umami receptors. Research carried out on the mGlu2‐4 heterodimer indicates that mGlu4-7TM is responsible for G protein activation, even if mGlu2 in the homodimer form can also activate G protein (Liu et al., 2017). Nevertheless, the TAS1R2 and TAS1R3 subunits can activate signal transduction in cell-based assays by themselves with specific ligands, suggesting that G protein activation could be mediated by each subunit for homodimer receptors, while in a heterodimer, the allosteric interaction leads only one 7TM to activate the G protein. Therefore, interaction between VFTs could promote activation of both 7TM domains in a symmetric or asymmetric way, as described for mGlu receptors (Koehl et al., 2019; Liu et al., 2017). More data will be needed to understand the multiple allosteric interactions and cooperativity between each VFT, CRD and 7TM domain in TAS1Rs. To achieve this goal, allosteric modulators and ligands well defined and characterized for their binding sites would be helpful.

## 4 Conclusion

In conclusion, we have shown that IMP, L-Glu and L-Asp are able to bind TAS1R1- and TAS1R3-VFT proteins with an affinity in the micromolar range. The modulator role of IMP, comparable with cyclamate, on the activation of the TAS1R3/TAS1R3 homodimer receptor and the heterodimeric TAS1R2/TAS1R3 was highlighted using cellular assays. We also showed that IMP potentiates TAS1R2/TAS1R3 receptor response to sweeteners, such as sucralose, neotame and cyclamate through TAS1R3-VFT binding. Thus, IMP and maybe other umami compounds, in cooperation with sweet-tasting compounds, contribute to enhancing the sweetness of sweetener blends used as food additives and can be considered as PAM for sweet taste. Additional studies using site-directed mutagenesis and molecular modelling are needed to identify the amino acid residues of TAS1R3-VFT involved in the binding of IMP and investigate the receptor allostery. These findings suggest more complex interactions at the receptor level between umami and sweet taste qualities and open the field to the development of new enhancers for sweetness.

## Funding

This research did not receive any specific grant from funding agencies in the public, commercial, or not-for-profit sectors.

## CRediT authorship contribution statement

**Christine Belloir:** Writing – review & editing, Writing – original draft, Visualization, Resources, Project administration, Methodology, Investigation, Funding acquisition, Formal analysis, Data curation, Conceptualization. **Lucie Moitrier:** Visualization, Investigation. **Adeline Karolkowski:** Writing – review & editing, Visualization. **Nicolas Poirier:** Visualization, Investigation. **Fabrice Neiers:** Writing – review & editing, Supervision. **Loïc Briand:** Writing – review & editing, Supervision, Project administration, Funding acquisition, Conceptualization.

## Declaration of competing interest

The authors declare that they have no known competing financial interests or personal relationships that could have appeared to influence the work reported in this paper.

## Data Availability

Data will be made available on request.

## Appendix A Supplementary data

Supplementary material Image 1
