# Supplementary material for: Inosine-5′-monophosphate interacts with the TAS1R3 subunit to enhance sweet taste detection
Source: Food Chem (Oxf). 2025 Feb 11;10:100246. doi: 10.1016/j.fochms.2025.100246 (PMC11872639; doi:10.1016/j.fochms.2025.100246)
Supplement: Supplementary file 1 — Supplementary material [file mmc1.docx]

# Supplementary data


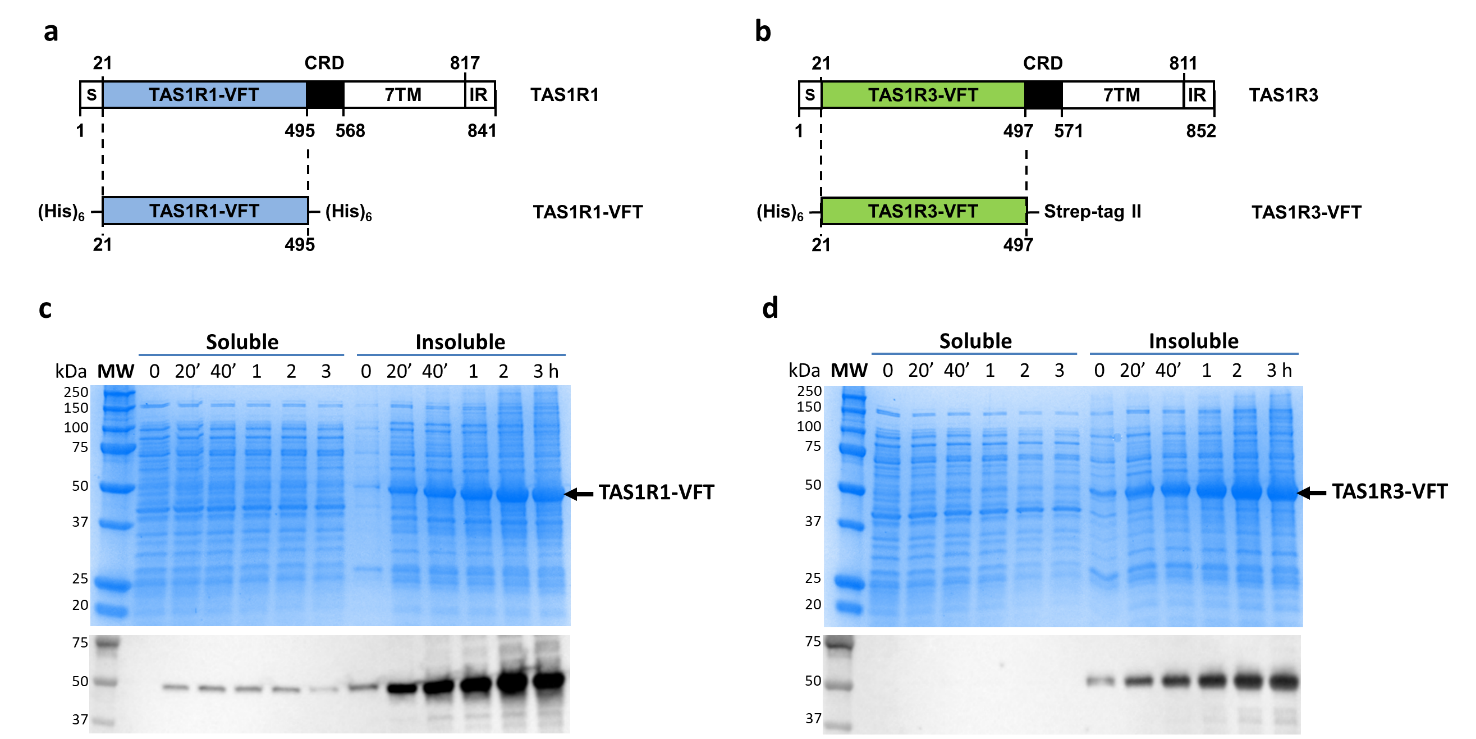


**Figure S1.** Strategy used for expression of TAS1R1- and TAS1R3-VFT proteins in bacteria. Schematic diagram of the expression construct pET28-TAS1R1-VFT (a) and pET28-TAS1R3-VFT (b). Length is presented according to its primary amino acid sequence deduced from TAS1R1 cDNA (GenBank ID: NM_177540) and TAS1R3 cDNA (GenBank ID: NM_152228). The numerical positions of amino acid residues for each subunit are indicated. The Venus Flytrap domain (VFT) was expressed independently from the 7-helix transmembrane domain (7TM) minus a short putative signal peptide (S), and the cysteine-rich domain (CRD). The double-tagged recombinant proteins expressed in this study are shown below. SDS–PAGE and Western Blot analysis of TAS1R1-VFT (c) and TAS1R3-VFT (d) expressed in *E. coli. BL21 (DE3)* cells transformed with pET28-TAS1R1-VFT or pET28-TAS1R3-VFT were induced with IPTG until 3 hours. Soluble and insoluble protein fractions (Inclusion bodies, IB) were separated and loaded onto 4-15 % SDS–polyacrylamide gel with size control of molecular mass markers (lane MW). Proteins were stained with Coomassie blue or subjected to transfer on PVDF membrane. The mouse anti-His primary antibody (Bio-Rad) diluted at 1/500 and the goat anti-mouse horseradish peroxidase-conjugated secondary antibody (1/25000) were used. The protein antibody complexes were detected using an ECL chemiluminescent kit and the Chemidoc XRS Imaging System (Bio-Rad). Position of TAS1R-VFTs is indicated by an arrow.


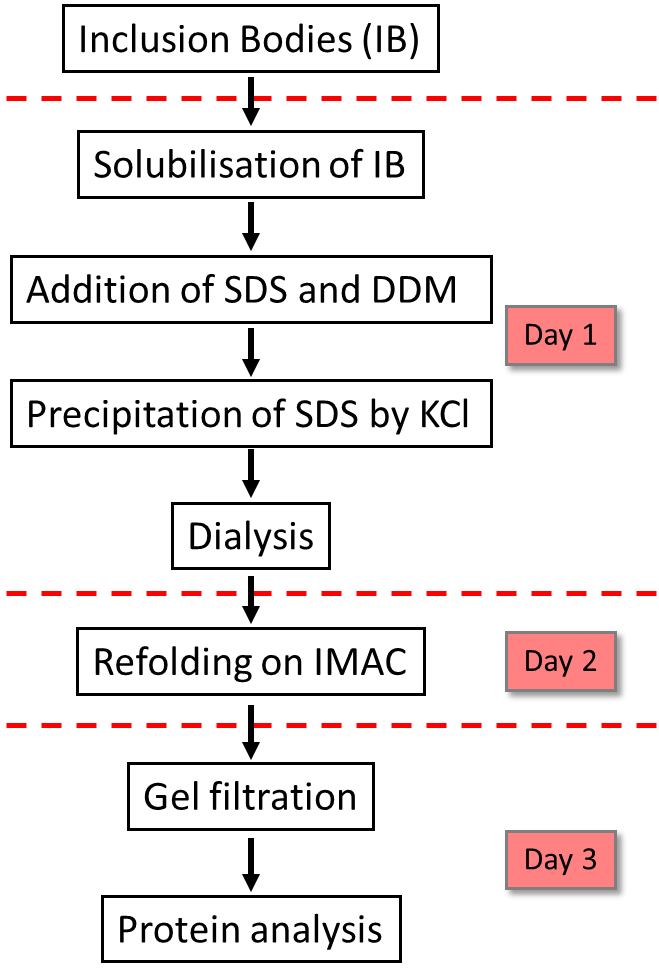


**Figure S2.** Outline of the strategy for the production of TAS1R-VFT proteins. Inclusion bodies (IB) were solubilized in lysis buffer containing 50 mM Tris-HCl pH 8.0, 0.6 % SDS, 8 M urea, 25 mM DTT and 0.1 mM DDM. After removal of urea by dialysis (50 mM Tris-HCl pH 8.0, 0.06 % SDS, 1 mM DTT and 0.1 mM DDM), the DDM concentration was adjusted to 5 mM followed by SDS precipitation by addition of KCl. The SDS precipitate was removed by centrifugation and the supernatant was dialyzed against dialysis buffer (50 mM Tris-HCl pH 8.0, 1 mM DTT and 0.1 mM DDM), then subjected to refolding on IMAC (Immobilized Metal Affinity Chromatography). Gel filtration was performed using a Superdex 200 10/300GL column.


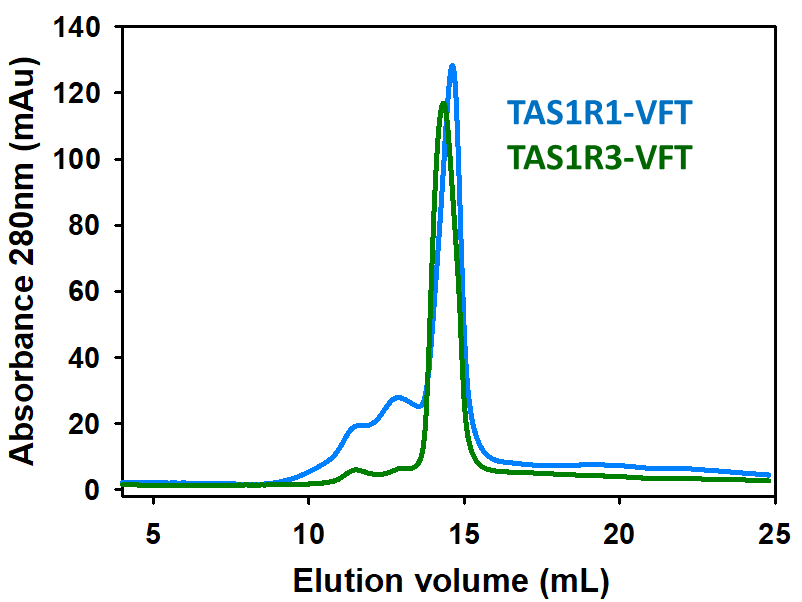


**Figure S3.** Gel filtration analysis of refolded TAS1R1- and TAS1R3-VFTs. The chromatography was performed using a Superdex 200 10/300GL column with 50 mM Tris-HCl pH 8.0, 150 mM NaCl, 1 mM DTT and 0.1 mM DDM as elution buffer.


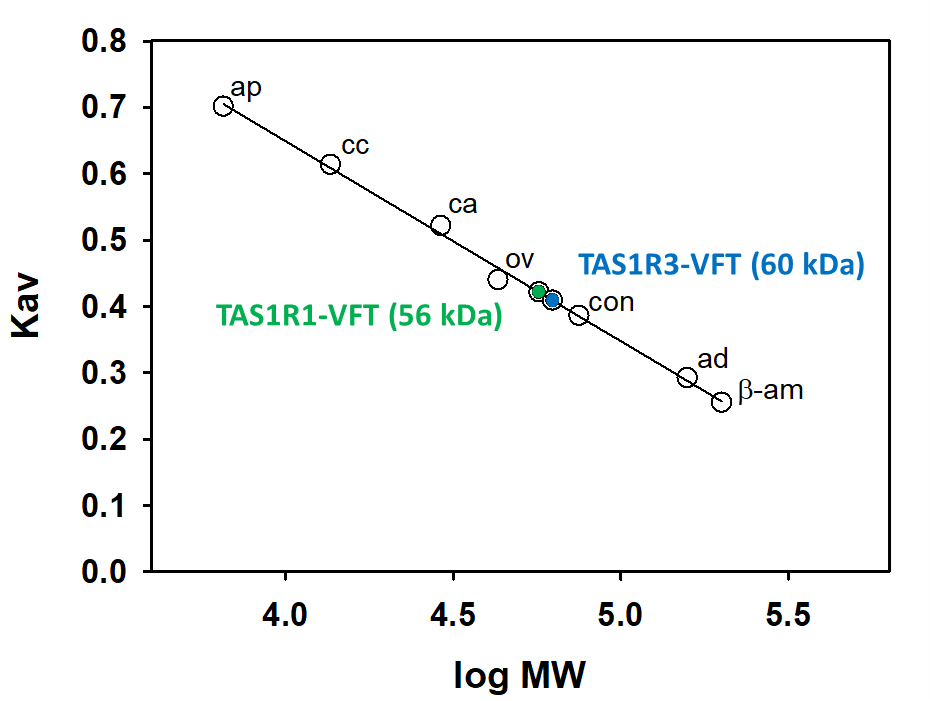


**Figure S4.** Gel filtration chromatography calibration. The calibration was performed using a Superdex increase 200 10/300GLcolumn (GE Healthcare). The column was equilibrated and eluted with 50 mM Tris-HCl pH 8.0, 1 mM DTT and 0.1 mM DDM. The calibration curve for the Superdex 200 column was established with β-amylase (β-am, 200 kDa), aldolase (ad, 158 kDa), conalbumin (con, 75 kDa), chicken egg ovalbumin (ov, 43 kDa), carbonic anhydrase (ca, 29 kDa), cytochrome C (cc, 12.4 kDa) and aprotinin (ap, 6.5 kDa). The estimated molecular mass of hTAS1R1- and hTAS1R3-VFTs are 56 and 60 kDa, respectively.


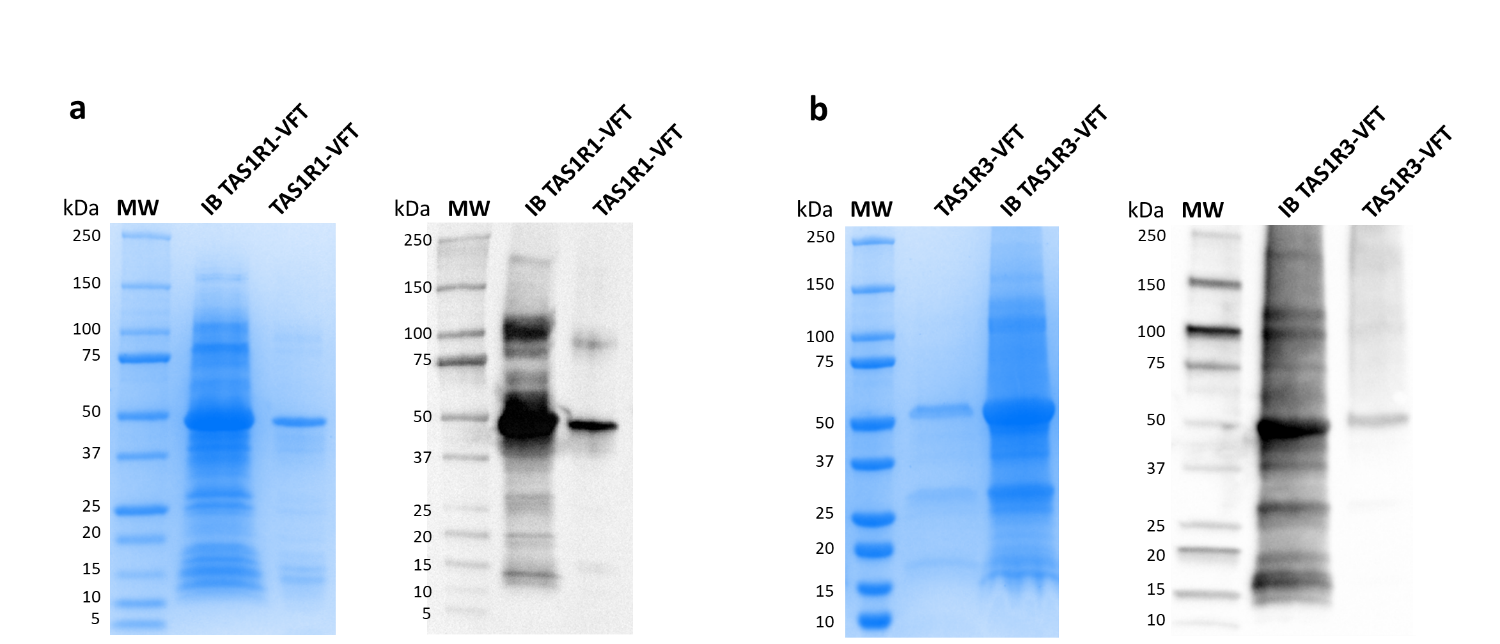


**Figure S5.** SDS-PAGE and Western blot analysis of purified inclusion bodies (IBs) of TAS1R1- (a) and TAS1R3-VFTs (b). The protein sample, inclusion bodies and purified TAS1R-VFTs after gel filtration were separated by 4-15 % SDS-PAGE and stained with Coomassie blue or analysed by Western blotting using the anti-His antibody. The molecular weight (MW) standards (Bio-Rad) are shown.


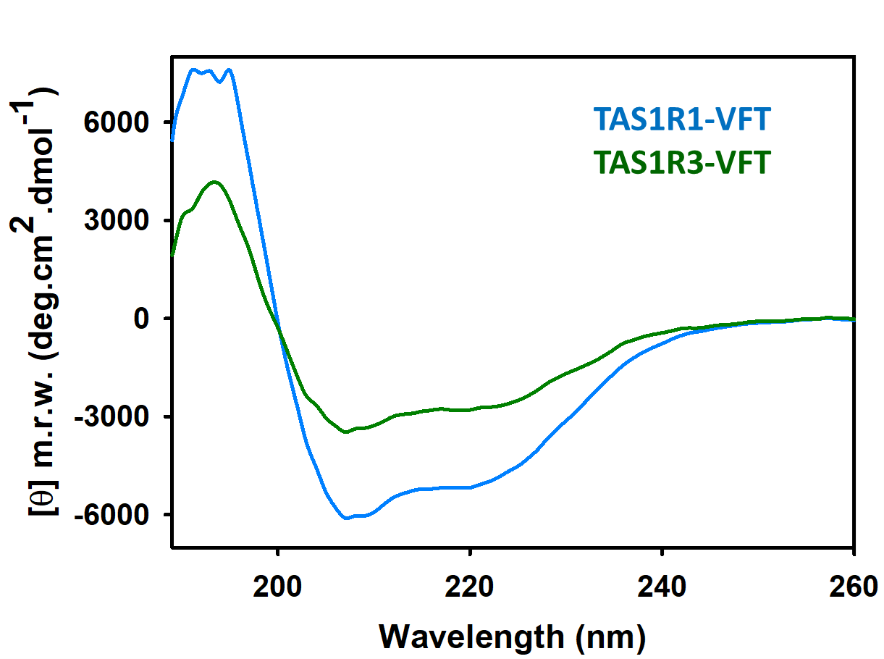


**Figure S6.** Secondary structure determination by circular dichroism. The far-UV CD spectra of purified TAS1R1-VFT (blue line) and TAS1R3-VFT (green line) were recorded after protein concentration. Light path: 0.01 cm. The spectra reveal the presence of a high content of α-helical secondary structures. The deconvolution of the CD spectra revealed that TAS1R1-VFT was composed of approximately 67 % α-helices and 10 % β-sheets, compared to 45 % α-helices and 12 % β-sheets for TAS1R3-VFT.


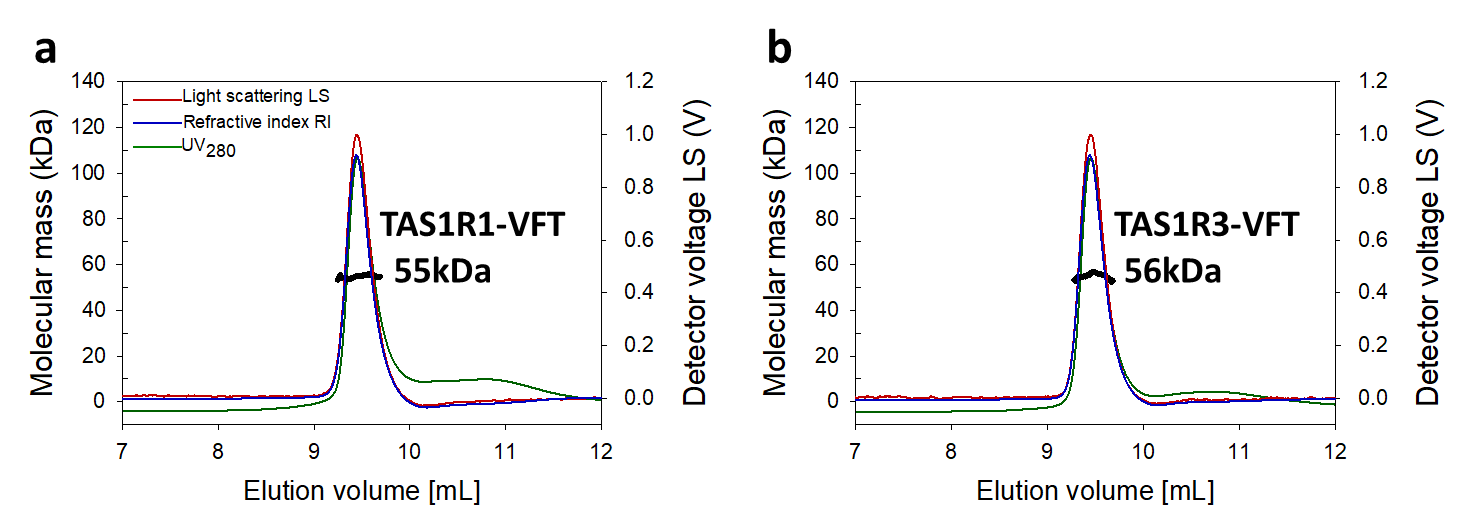


**Figure S7.** SEC-MALS analysis of TAS1R-VFT proteins. Peak observed in the previous SEC analysis (see Figure S3) were subjected to a weight analysis using SEC coupled to a triple-angle light scattering detector, a UV detector (280 nm) and a differential refractometer with gel filtration column KW-803 (Shodex) in buffer containing 50 mM Tris-HCl pH 8.0, 150 mM NaCl and 0.1 mM DDM. The numbers printed next to the peaks are the average MW values (kDa) for the entire peak. One main monomeric form with a fitted molecular mass of 55 (a) and 56 kDa (b) was observed for TAS1R1-VFT and TAS1R3-VFT, respectively.


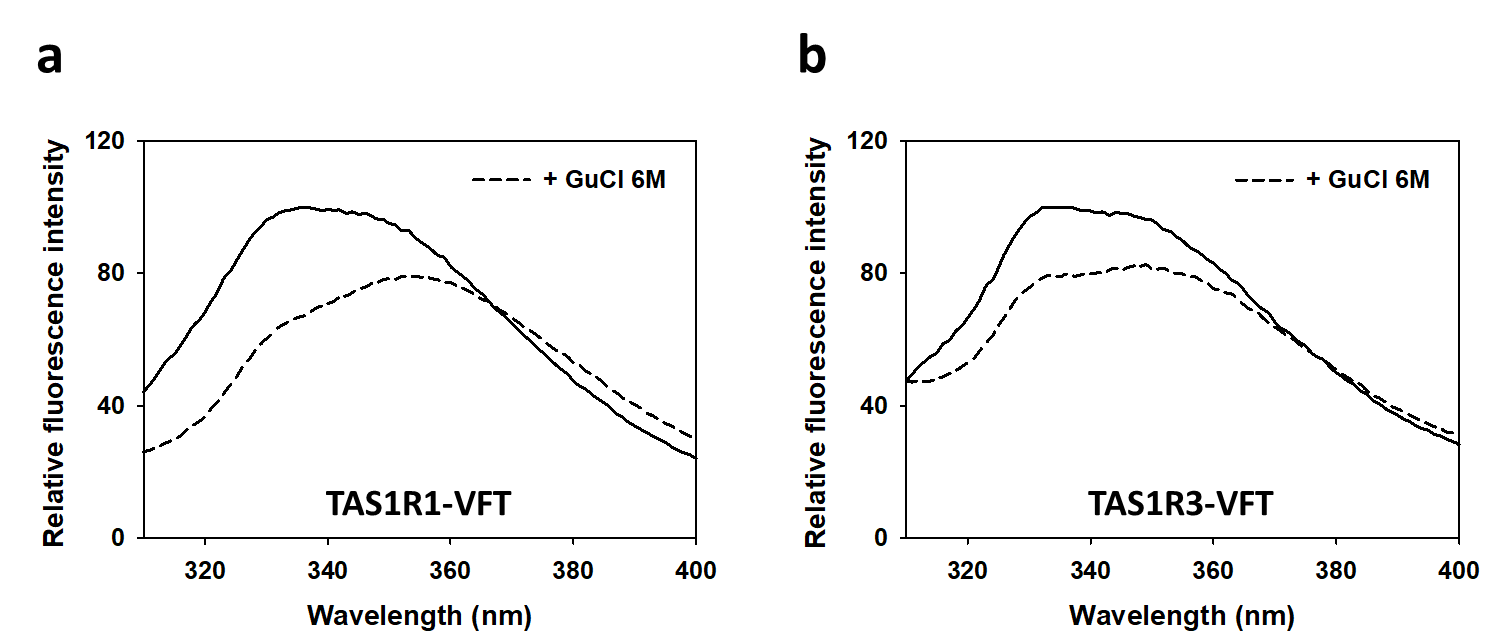


**Figure S8.** Chemical denaturation of TAS1R-VFT proteins followed by intrinsic tryptophan fluorescence. Addition of 6 M guanidinium chloride (GuCl) induced a red shift of 17 nm of the emission maximum and 40 % decrease in tryptophan fluorescence intensity for TAS1R1-VFT (a) and a red shift of 16 nm of the emission maximum and 21 % decrease in fluorescence intensity for TAS1R3-VFT (b). These data confirmed that the refolded TAS1R-VFT proteins were properly folded.
